# New Proposal for Inverse Algorithm Enhancing Noise Robust Eddy-Current Non-Destructive Evaluation

**DOI:** 10.3390/s20195548

**Published:** 2020-09-28

**Authors:** Milan Smetana, Lukas Behun, Daniela Gombarska, Ladislav Janousek

**Affiliations:** Department of Electromagnetic and Biomedical Engineering, Faculty of Electrical Engineering and Information Technology, University of Zilina, Univerzitna 1, 010 26 Zilina, Slovakia; milan.smetana@feit.uniza.sk (M.S.); behun@esys.sk (L.B.); daniela.gombarska@feit.uniza.sk (D.G.)

**Keywords:** non-destructive evaluation, eddy current, 3D sensing, inverse problem, wavelet transform, principal component analysis, neural network

## Abstract

Solution of inverse problem in eddy-current non-destructive evaluation of material defects is concerned in this study. A new inverse algorithm incorporating three methods is proposed. The wavelet transform of sensed eddy-current responses complemented by the principal component analysis and followed by the neural network classification are employed for this purpose. The goal is to increase the noise robustness of the evaluation. The proposed inverse algorithm is tested using real eddy-current response data gained from artificial electro-discharge machined notches made in austenitic stainless-steel biomaterial. Eddy-current responses due to the material defects are acquired using a newly developed eddy-current probe that senses separately three spatial components of the perturbed electromagnetic field. The presented results clearly show that the error in evaluation of material defect depth using the proposed algorithm is less than 10% even when the signal-to-noise ratio is as high as 10 dB.

## 1. Introduction

Electromagnetic non-destructive evaluation of conductive materials is currently used in various strategy sectors, such as aviation, nuclear, petrochemical, biomedicine, and many other industries. There are several methods that utilize the interaction of the electromagnetic field with a conductive structure for the purpose. One of the frequently used methods is the eddy current testing (ECT). It originates from the electromagnetic induction phenomena and the principle of ECT underlies in the interaction of induced eddy currents with structure of an examined body. This method is a powerful tool for non-destructive evaluation of material discontinuities. Its main advantages include high sensitivity, high inspection speed, and versatility. The main limitation is the possibility for investigating of surface and close subsurface defects only. This limitation results directly from the physical principle of the method as the induced eddy currents are strongly attenuated along a material depth. In the non-destructive evaluation, two approaches are defined: forward and inverse problem solution. Forward problem is a direct approach of acquiring responses from a defect by experimental measurements or numerical simulations. The solution of inverse problem involves the identification of the defect under investigation from the responses; for example, identification of its dimensions, geometry, shape, and orientation. In general, solving of the inverse problem is always relatively difficult. Many research teams, mostly from the academic environment, deal with this issue using the ECT method. ECT is a relative method and the inverse problem is thus ill-posed [1]. Characterization of a detected defect from ECT response signals is quite a challenge. Various mathematical procedures are being sought and used with the aim of partial improvements of preciseness and reliability. Scientific works are focused mainly on reducing uncertainty in estimating parameters of defect geometry, especially by applying adaptive Monte Carlo method [2], metamodels-based Markov–Chain–Monte-Carlo [3], Genetic Algorithm [4], Neural Networks [5], Particle Swarn Optimization [6], and others [7,8]. Many published papers address the effect of a defect structure on response signals and then optimize 3D defects [9]. A separate chapter is represented by non-iterative methods and methods for eliminating artefacts such as lift-off variation [10]. The main goal is to correctly estimate the defect geometry at first. It is then possible to reconstruct the predicted shape of the defect using appropriate mathematical procedures. However, all these procedures are considerably computationally time-consuming. If one compares the calculation times for defects of defined shape (electric-discharge-machined, EDM) vs. corrosion defects (stress-corrosion-cracking, SCC), the computational-time difference can reach up to hundred-times under the same conditions. There is currently no algorithm that can provide such real-time results. In addition to the calculation time criterion, other criteria are present: accuracy and unambiguity of calculation, permissible error, resistance and robustness to noise, and others [11,12]. The noise resistance is a very important criterion since the noise greatly increases the error of the defect parameters estimation. The origin of the noise is mainly caused by various artefacts: lift-off variation, superimposed measurement noise, too low probe resolution, and ambient electromagnetic disturbances, [13,14]. 

The paper focuses on increasing noise robustness in ECT inverse problem solution. A new inverse algorithm is proposed for the purpose. The algorithm combines three methods: wavelet transform, principal component analysis, and neural network. Moreover, newly developed ECT sensor is employed for the inspection. It is experimentally demonstrated that the error of a detected crack depth evaluation from the ECT response signals is quite low even when the signal to noise ratio is as high as 10 dB.

## 2. New Inverse Algorithm

The solution of ECT inverse problem and successful reconstruction of detected flaw properties or its character strongly depend on the chosen method/s. All conventionally used methods are dependent on database of known eddy-current responses and corresponding crack geometry including dimensions or on optimization and solution of forward problems using numerical means. The contemporary main aim of the methods’ development is to obtain relevant crack geometry and/or to visualize 3D crack profile in real time with high preciseness. The preciseness of reconstruction is further deteriorated by presence of noise in the measured signal. Since the nature of the present noise is a stochastic one and it comes from multiple sources, the use of noise robust inverse algorithm is thus essential. The inverse problem solution proposed in the paper applies wavelet transform (WT) on measured signal complemented by principal component analysis (PCA) and followed by neural network (NN) classification. In the ECT field, the most important informations are depth and length of a detected crack [9,13,14]. Specifically, the crack depth is the most critical parameter from the structural integrity point of view. A crack width is not reconstructed in this paper as this parameter is not important from the structural integrity point of view. Artificial as well as natural cracks are very narrow, and their opening is on the level of tenth of millimeter. Moreover, this parameter (in its natural range) almost does not influence the ECT responses.

**Figure 1:**
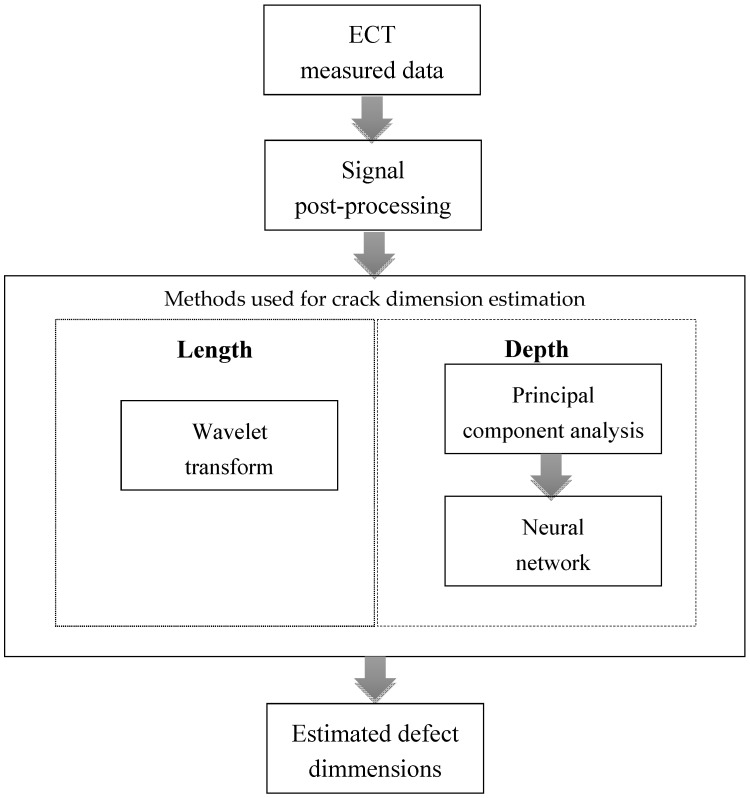
Flowchart of proposed new inverse algorithm.

It should be noted that the ECT is the indirect method and the ECT responses are integral ones. Several methods have been examined in other to develop noise robust algorithm for a crack dimension estimation from ECT responses. The combination of WT, PCA, and NN shows promising results. The flowchart of the proposed new inverse algorithm is shown in Figure 1. The WT is employed for a crack length estimation. Due to generally known limitations of ECT, a crack depth estimation is more difficult. It is possible to determine a crack depth based on the amplitude of a measured signal—the greater the depth, the higher the amplitude of the measured signal. However, this method of estimation is greatly influenced by the lift-off, noise, skin-effect, etc. Therefore, the depth determination from the signal amplitude is often erroneous and inaccurate. For this reason, artificial intelligence is employed in this paper to tackle this issue. The amount of data is reduced at first using PCA. The depth of a defect is then estimated using trained NN. The methods employed in the proposed new algorithm are explained below in details.

### 2.1. Wavelet Transform

Measured ECT signals are of non-stationary character. To identify the frequency components of non-stationary signals various time–frequency analysis methods have been developed including linear and bilinear time–frequency representations, adaptive parametric and non-parametric time–frequency analysis, and more. Linear time–frequency methods decompose signals into a weighted sum of a series of bases localized in both time and frequency domains. The resolution in time–frequency domain, however, is governed by the Heisenberg uncertainty principle: the time localization and frequency resolution cannot reach their highest levels together. To match the complex structure of measured non-stationary signal, the WT is chosen here. The WT as time–scale analysis method gives an effective tool for analyzing self-similar signals. As the basis, the WT employs wavelets and adds a scale variable in the inner product transform to the time variable. It has a good frequency resolution for lower frequencies, which does not further work for higher frequencies. For higher frequency components, WT has a better time localization, but a lower frequency resolution. Thus, for lower frequency components the time localization is worse. To date, various types of wavelet basis have been proposed. However, the question of choice remains an open issue. Choosing a suitable one among all to match the signal structure requires experiences and generally accepted effective methods do not exist. Continuous WT of any energy limited signal *x*(*t*) can be defined as:

(1) $$X_{W}\left(s,b\right)=\frac{1}{\sqrt{s}}\int_{-\infty }^{\infty }x\left(t\right)\psi^{*}\left(\frac{t-b}{s}\right)dt$$

where *ψ*(*t*) is the basis mother wavelet and the transform is derived by dilating with scale *s* and translating by the shift parameter *b*. Energy conservation of transform is maintained by the normalization factor 1/√*s*. The Haar wavelet is chosen as the base mother wavelet for processing the measured signals here, because such an application requires the usage of non-symmetrical wavelets.

The WT is used to detect changes in signal amplitude gradient and to estimate scalograms for evaluation of a detected defect dimension, its length in this case. Whilst the length is estimated from the amplitude gradient changes in columns and rows of measured signal matrix, the depth estimation requires an adaptation. 

An important step in the use of WT is the appropriate selection of the mother wavelet, which depends on the specific use of WT. The selection of the wavelet is conditioned by monitoring the changes in the gradient. Asymmetric wavelets are suitable for detecting changes in the gradient of the signal under investigation, so the Haar wavelet is chosen. Using Haar wavelet changes in the gradient in the measured signal are detected and detecting the changes the information about the defect and its dimensions is obtained. The mother wavelet is used to calculate the WT coefficients from the matrix of input data.

**Figure 2:**
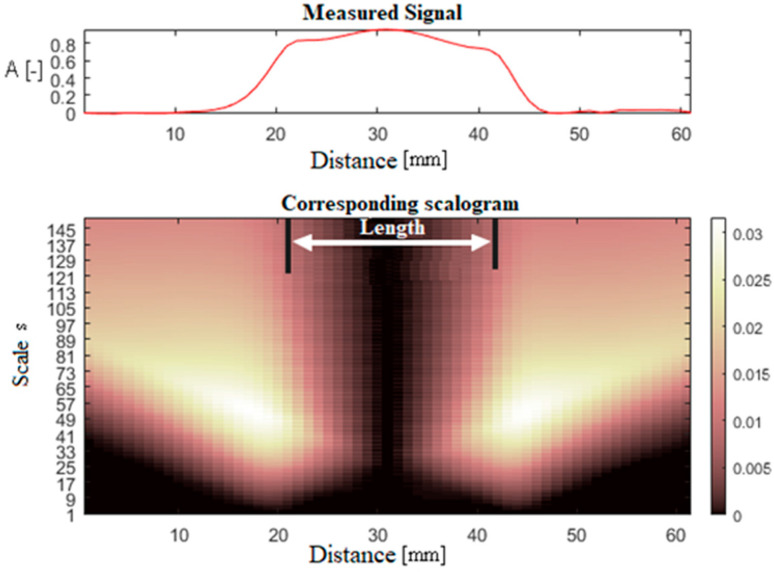
Scalograms for one column of the input data matrix-sample scalogram.

**Figure 3:**
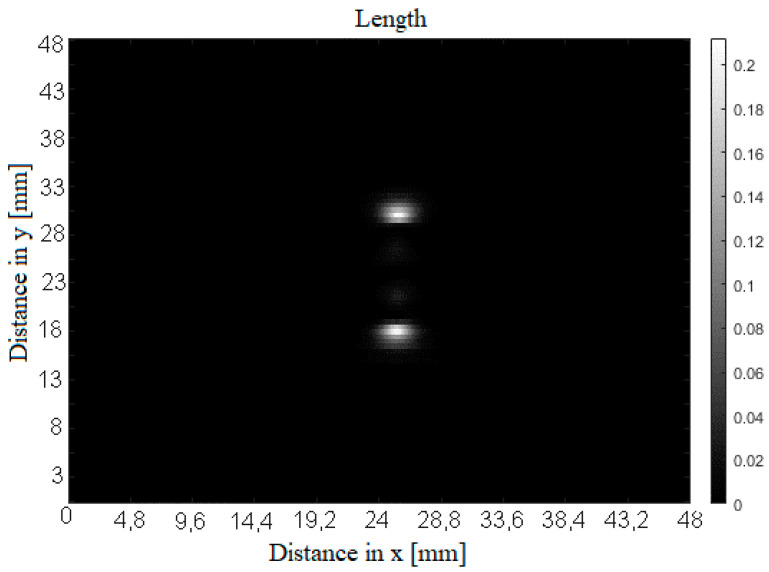
Matrix containing individual column vectors of WT coefficients.

The convolution process is performed by individual columns of the input matrix to determine the length of the defect. To illustrate the resulting scalogram, the coefficient values for one column are shown in Figure 2. The vector for the value of the scale *s* = 150 is subsequently extracted from the scalogram. Such a scale represents information about slowly changing signal details, i.e., low frequencies. Subsequently, a matrix is created in which these vectors of WT coefficients are written. Figure 3 displays an example of such matrix converted into a grayscale image. The bright spots specify the edges of the defect for the case of length determination.

### 2.2. Principal Component Analysis

Usage of the signal amplitude does not work well for the estimation of a detected defect depth; it means the defect dimension in direction towards material thickness. PCA algorithm is adopted for the data processing prior the estimation of a detected defect depth. The first step is to normalize input data by scaling the specific properties $x_{j}^{\left(i\right)}$ by their mean values $s_{j}$:

(2) $$s_{j}=\frac{1}{m}\overset{m}{\underset{i=1}{\sum }}x_{j}^{\left(i\right)}$$

where *j* is the index of specific property. The required reduction of data dimension by one is achieved by means of covariance matrix and eigenvectors, where the covariance matrix is:

(3) $$\sum =\frac{1}{m}\overset{m}{\underset{i=1}{\sum }}\left(x^{\left(i\right)}\right){\left(x^{\left(i\right)}\right)}^{T}$$

The eigenvector of the covariance matrix is computed by means of singular values decomposition (SVD). The PCA data reduction for the neural network input decreases data from 10^5^ to 10^3^. This approach significantly reduces computational demands of classification problem. The output data from PCA are fed to the neural network for further classification.

### 2.3. Neural Network

**Figure 4:**
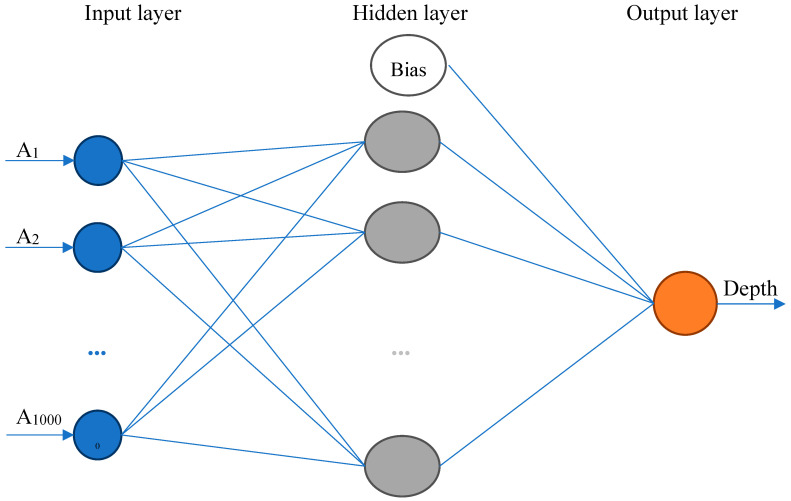
Arrangement of the neural network.

A NN employed for a crack depth estimation uses back-propagation algorithm with forward propagation of input data, backward propagation of error, and consequent changes in input neuron weight values. The real measured data for each defect with known geometry are applied as the training sets and the applied training function is Bayesian regularization based on Levenberg–Marquardt optimization, [3,9,10]. The neural network consists of an input, a hidden and an output layer. The number of input neurons depends on the length of the eigenvectors. The hidden layer consists of ten neurons, and the output layer, since the output is the defect depth value, is formed by one neuron. The activation functions between the input and hidden layer is sigmoid and between hidden and output layer is linear function. The simple neural network model is shown in Figure 4. In the present work, the input layer has 1000 input neurons, which correspond to measured samples in one row. The output layer value represents an estimated parameter-a depth dimension of a detected defect. To achieve faster convergence compared to standard back propagation neural network, the Levenberg–Marquardt algorithm is used. The Levenberg–Marquardt algorithm uses approximation to the Hessian matrix instead of computing it directly. In the method, the Marquardt adjustment parameter *μ* is introduced. The parameter *μ* is decreased after each successful step in order to reduce the performance function at each iteration. Besides that, to avoid overfitting during neural network training, the Bayesian approach is used. Typically, the training aims to reduce the sum of squared errors. The Bayesian framework also considers the sum of squares of the network weight and the objective function. It also considers the weights of the network to be random variables. According to the Bayes’ rule, the probability distribution can be written as:

(4) $$P\left(w|D,\alpha ,\beta ,\eta \right)=\frac{P\left(D|w,\beta ,\eta \right)P\left(w|\alpha ,\eta \right)}{P\left(D|\alpha ,\beta ,\eta \right)}$$

where *D* corresponds to the input–output data set, *η* denotes the network model and architecture, *w* are the network weights, and *α*, *β* are objective function parameters. *P*(*w*|*α*,*η*) is the prior distribution derived of the knowledge about the weights before any data are collected, *P*(*D*|*w*,*β*,*η*) is the probability of the data occurring given the weights *w*. *P*(*D*|*α*,*β*,*η*) is the normalized factor which guarantees that the probability is 1. The network is trained using experimental dataset of 70 similar EDM notches.

## 3. Experimental Setup

**Figure 5:**
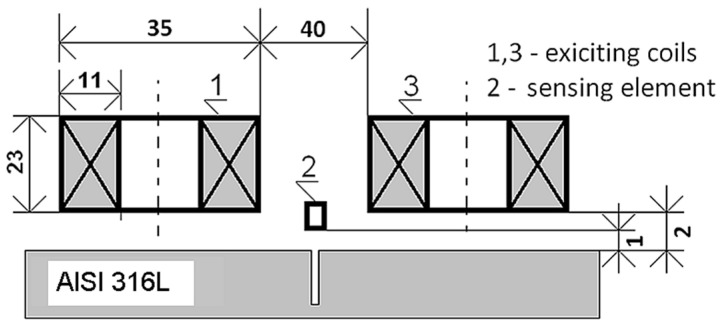
Arrangement of the eddy current testing (ECT) probe over a specimen (dimensions are in mm).

A modular ECT probe developed and constructed by authors is employed to excite eddy currents in an inspected specimen and to acquire the eddy current responses due to material discontinuities. Schematic arrangement of the ECT probe over a specimen is shown in Figure 5 (dimensions are given in mm).

The ECT probe consists of two identical exciting circular coils positioned apart from each other and oriented normally regarding the surface of an inspected plate specimen. The coils are connected in series, but magnetically opposite to induce uniformly distributed eddy currents in the plate. The exciting coils are supplied from a harmonic source with a frequency of 1 kHz and the current density 1 A/mm^2^. A detection system (sensing element) of the probe is composed of a fluxgate magnetometer. Three spatial components of the perturbed magnetic flux density field are acquired during a crack inspection. The detection system is located in a centre between the exciting coils to gain high sensitivity as the direct coupling between the exciting coils and the sensing element is minimal at this position.

**Figure 6:**
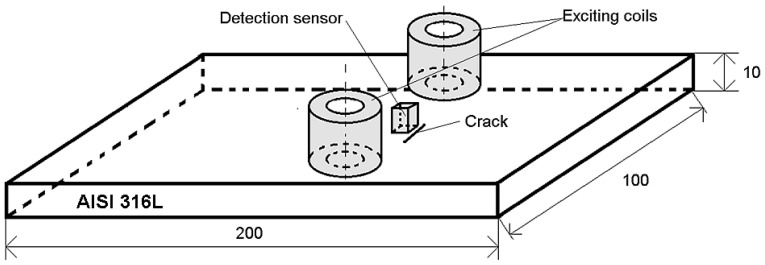
Spatial configuration of the plate specimen and the ECT probe (dimensions are in mm).

A non-magnetic conductive plate specimen with a thickness of 10 mm made from the stainless steel AISI316L is inspected in this study, as shown in Figure 6 (dimensions are given in mm). The material has the conductivity of *σ* = 1.4 MS/m and the relative permeability of *μ*_r_ = 1.

Four electro-discharge machined (EDM) notches of cuboid shape are introduced into the plate specimen. The dimensions of the EDM notches (cracks) are summarized in Table 1. The dimensions are denoted as *l*_c_—crack length; *w*_c_—crack width (opening); and *d*_c_—crack depth. One can see that the EDM notches differ in their lengths and depths. 

A precise 3-axial mechanical positioning system, so called XYZ stage, is employed to precisely position the ECT probe over a surface of the inspected body and to provide prescribed movement of the probe over the surface. Clearance between the probe bottom and the plate surface, so called lift-off, is kept contact during the whole inspection on a value of 1 mm. Two-dimensional scanning is performed over the cracked surface from the near side. The scanned area over each cracked region is 48 × 48 mm^2^. The probe moves smoothly along the crack length while the crack centre corresponds with a centre of the scanned area. The number of samples in one row is 1000 and the area of scanning is divided into 100 rows. The real and imaginary parts of all three spatial components of the perturbed magnetic flux density vector are sensed and recorded during the inspection of each crack. The XYZ stage control and the data acquisition are done under the LabVIEW environment.

## 4. Results and Discussions

**Figure 7:**
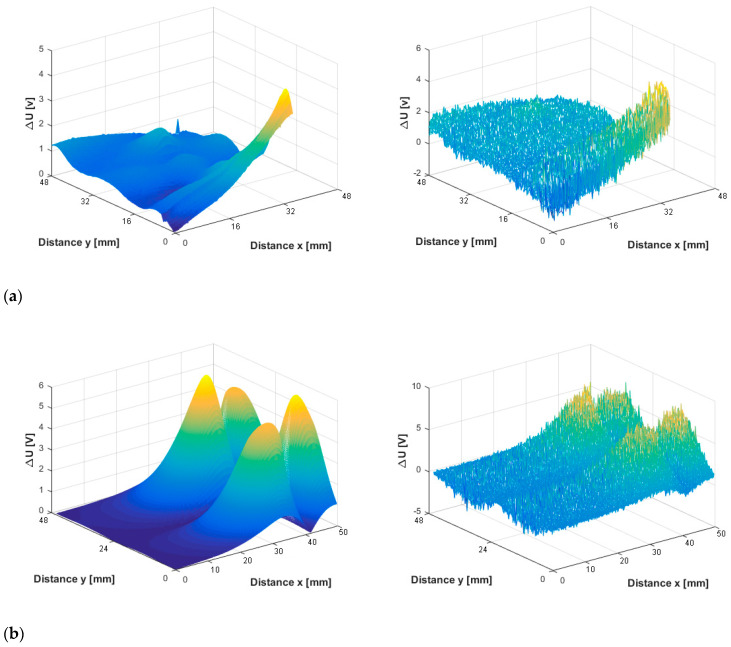

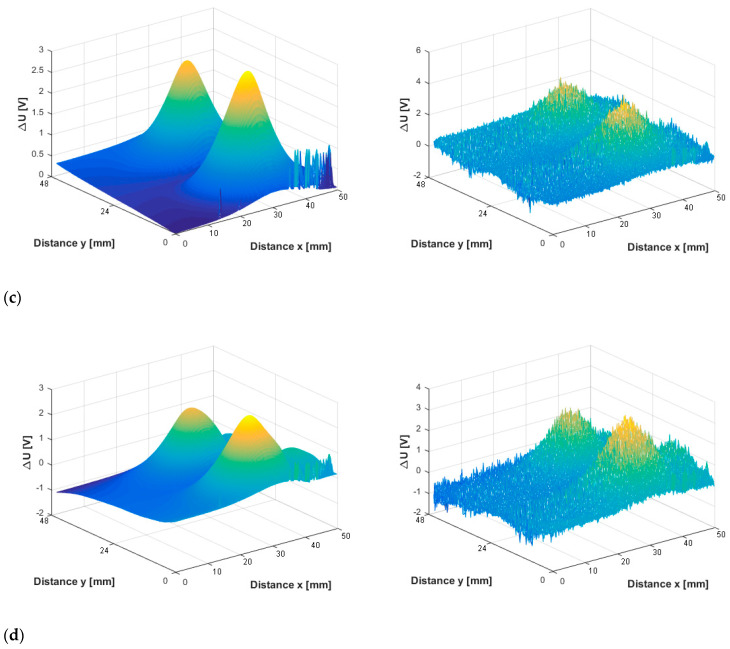
Eddy current responses due to an EDM notch measured during 2-dimensional scanning over a cracked region.

Four EDM notches introduced in the AISI 3016L plate specimen are inspected using the ECT probe according to the explanation provided in the previous section. Example of the sensed eddy current responses from one crack are presented in Figure 7. The Y-axis of the plots shows sensed voltage difference at the terminals of the fluxgate sensor and it is directly proportional to the measured perturbed magnetic flux density value. All three spatial components of the magnetic flux density vector are shown in corresponding rows of the figure: (Figure 7a) the X component of the perturbed magnetic flux density vector; (Figure 7b) the Y component of the perturbed magnetic flux density vector; and (Figure 7c) the Z component of the perturbed magnetic flux density vector), while the absolute value of the magnetic flux density is shown in the last row (Figure 7d). The sensed signals are shown in the left column. An additional white noise is artificially generated via waveform generator with different signal to noise ratio (SNR) values, i.e., 3 dB, 6 dB, and 10 dB, and added to the measured signal. The right column in Figure 7 shows corresponding signals deteriorated by the noise with SNR of 10 dB.

Crack dimensions are reconstructed based on the proposed inverse algorithm presented in the Section 2. The sensed responses as well as the ones with the added white noise of different SNR levels—3, 6, and 10 dB are used as input signals for the reconstruction algorithm, separately.

**Figure 8:**
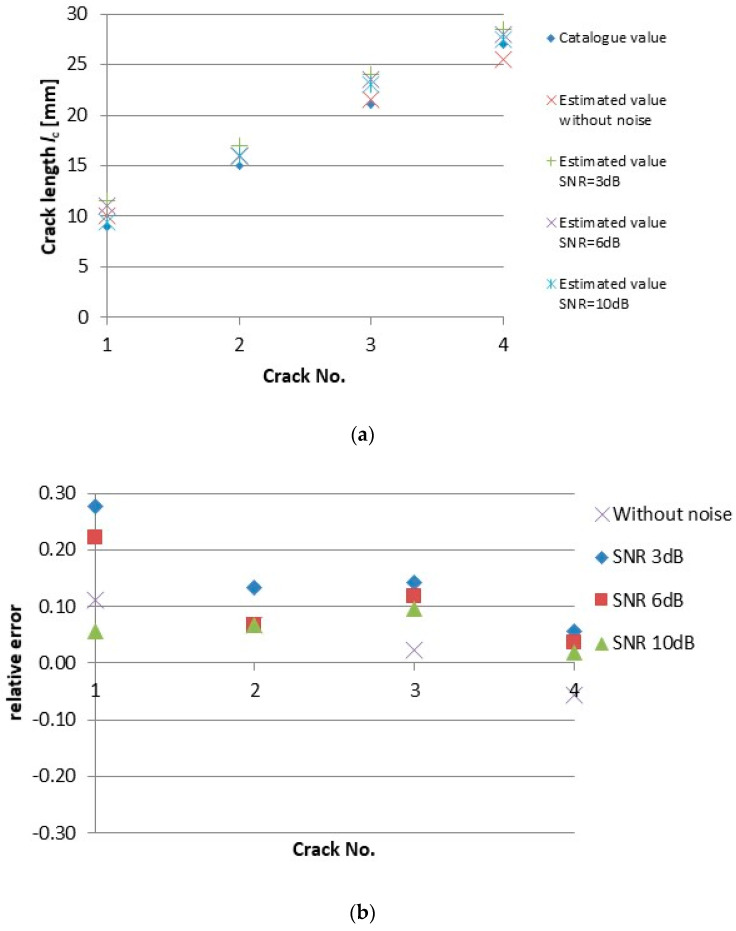
Results of the crack length *l*_c_ reconstruction from the ECT responses with different SNR: (**a**) estimated absolute values; (**b**) relative error of estimation.

The results of the crack length *l*_c_ reconstruction are reported in Table 2 for different noise levels. Data presented in column 0 dB are the ones reconstructed from the measured signal directly without any artificial noise added. Graphical representation of the results together with the relative error of reconstruction are shown in Figure 8. One can observe that in case of noisy signals the crack length is overestimated what is safe side from the non-destructive evaluation point of view. The estimation error is quite high in case of the crack No. 1, i.e., short and shallow crack. It is caused by large dimensions of the ECT probe employed for inspection and its sensitivity capabilities. The length of three other cracks, i.e., the longer (and deeper) ones, is estimated with quite good precision, especially the one of crack No. 4. It is important to highlight that the results prove that the proposed inversion algorithm is quite robust against the noise.

**Figure 9:**
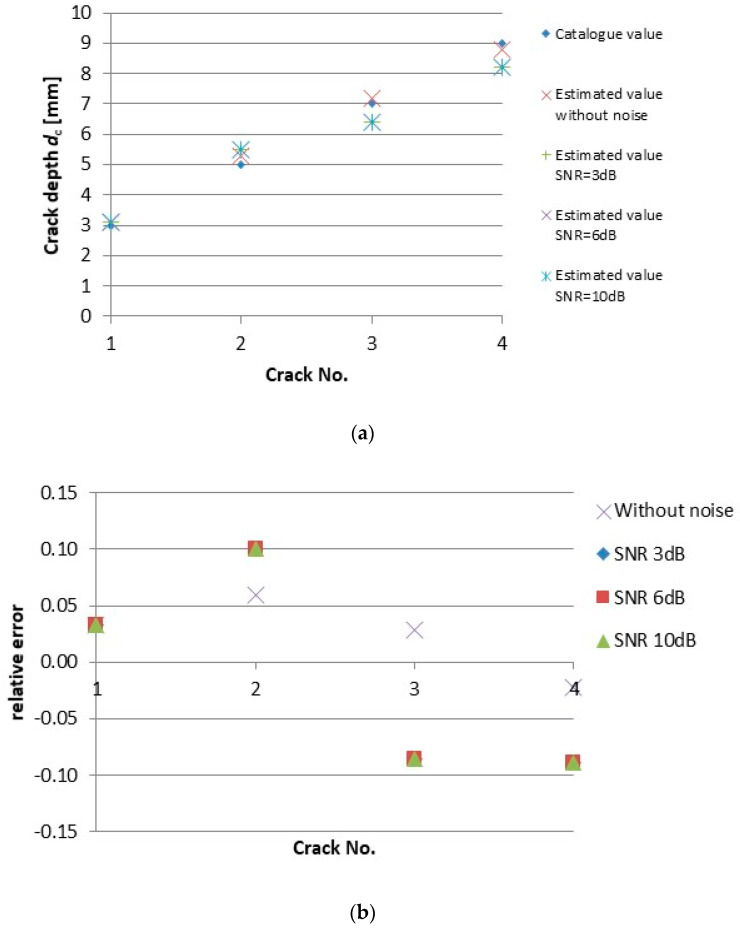
Results of the crack depth *d*_c_ reconstruction from the ECT responses with different SNR: (**a**) estimated absolute values; (**b**) relative error of estimation.

Precise estimation of a detected crack depth is more critical issue in non-destructive evaluation comparing to a crack length. The crack depth *d*_c_ reconstruction results are reported in Table 3 and in Figure 9. Figure 9 displays absolute values of the estimated depth of respective cracks as well as the relative error of reconstruction for each crack and each SNR. As it can be seen, the preciseness of the depth estimation is quite high, and the noise does not have almost any influence on the estimation results. The depth of shallower cracks is slightly overestimated, while the estimated values are a little bit lower than the real ones for the deeper cracks. This corresponds to the depth resolution of the probe itself and the eddy current attenuation along material thickness.

The experimental investigation and presented results clearly proved that the proposed new inverse algorithm presented in the paper is quite robust against the noise.

## 5. Conclusions

A new algorithm for noise-robust inverse analyses of eddy-current responses in non-destructive evaluation was presented in the paper. The algorithm employs wavelet transformation to estimate a length of a detected crack from the sensed eddy-current responses. The procedure continues with the principal component analysis for data reduction and finally the crack depth is estimated using the neural network. An eddy-current probe newly developed by authors is used for the non-destructive inspection. The probe drives eddy-currents with uniform distribution in an inspected object. The eddy-current responses are sensed using a magnetic sensor. All three spatial components of perturbed magnetic flux density vector are acquired during the inspection. Two-dimensional scanning of the probe over a cracked surface is performed using XYZ stage from the near side. A plate specimen with four electro-discharge machined notches was inspected in this study. The sensed responses were further deteriorated by noise of different levels. Dimensions of the notches were estimated from the sensed responses using the proposed algorithm. An artificial noise of different levels was added to the measured responses in order to evaluate noise-robustness of the developed inverse algorithm. It was presented that the estimated crack depth differs by less than 10% from the real ones even when the signal to noise ratio level is 10 dB. The obtained results demonstrated that the noise level in the investigated range almost do not have any impact on the preciseness of the estimation. 

## Figures and Tables

**Table 1:** Dimensions of the electro-discharge machined (EDM) notches.

Crack No.	*l*_c_ [mm]	*w*_c_ [mm]	*d*_c_ [mm]
1	9	0.20	3
2	15	0.25	5
3	21	0.25	7
4	27	0.25	9

**Table 2:** Values of the reconstructed crack length *l*_c_ [mm] from the ECT responses with different Signal to Noise Ratio (SNR).

Crack No.	Real Crack Length	SNR [dB]			
		0	3	6	10
1	9.0	10.0	11.5	11.0	9.5
2	15.0	16.0	17.0	16.0	16.0
3	21.0	21.5	24.0	23.5	23.0
4	27.0	25.5	28.5	28.0	27.5

**Table 3:** Values of the reconstructed crack depth *d*_c_ from the ECT responses with different SNR.

Crack No.	Real Crack Depth	SNR [dB]			
		0	3	6	10
1	3.0	3.1	3.1	3.1	3.1
2	5.0	5.3	5.5	5.5	5.5
3	7.0	7.2	6.4	6.4	6.4
4	9.0	8.8	8.2	8.2	8.2
